# *Vibrio cholerae* O6 gastroenteritis in a patient with lupus nephritis – a report from coastal Karnataka, South India

**DOI:** 10.1099/jmmcr.0.005171

**Published:** 2018-12-19

**Authors:** Mamatha Ballal, Vignesh Shetty, Sohan Rodney Bangera, Asish Mukhopadhyay, Goutam Chowdhury, Prosenjit Samanta, Akshata Prabhu, H. C. Anusha

**Affiliations:** ^1^​ Enteric Diseases Division, Central Research Lab, Kasturba Medical College, Manipal University, Manipal, Karnataka, India; ^2^​ Division of Bacteriology, National Institute of Cholera and Enteric Diseases, Beliaghata, Kolkata, India

**Keywords:** lupus nephritis, *Vibrio cholerae* O6, non-O1/non-O139 vibrios, non-agglutinating vibrios, acute gastroenteritis, fever, ciprofloxacin

## Abstract

**Introduction:**

*Vibrio cholerae* O1 strains are responsible for pandemics of cholera and major epidemics in the world. All the remaining *V. cholerae* non-O1/non-O139 strains are less virulent and are responsible for sporadic cases of gastroenteritis. These non-O1/non-O139 serogroups have more than 200 somatic antigens, and mostly lack cholera toxin and toxin co-regulated pilus encoding genes. Toxigenic and non-toxigenic non-O1/non-O139 *V. cholerae* have caused several diarrhoeal outbreaks in India and other countries. Acute gastroenteritis is the typical clinical sign and symptom of non-O1/non-O139 *V. cholerae* infection for both periodical and outbreak cases; in contrast, these *V. cholerae* are rarely associated with extraintestinal infections.

**Case presentation:**

Here, we present a case of a 27-year-old female with underlying kidney disease (lupus nephritis) presenting with loose stools, vomiting and fever. *V. cholerae* O6 was isolated from a faecal sample, which was positive for *hlyA* and the type III secretion system. The present case is, to the best of our knowledge, the first such case to be reported from South India.

**Conclusion:**

The *V. cholerae* O6 associated with autoimmune disease in the present study demonstrates the role of this pathogen in acute gastroenteritis, and if it is left undiagnosed it can lead to septicaemia and other complications. The pathogenic mechanisms of non-O1/non-O139 *V. cholerae* are multivariate, virulence factors being naturally present in these strains. Therefore, further epidemiological studies are necessary to determine the virulence factors and their pathogenic mechanisms. Non-O1/non-O139 *V. cholerae* can undoubtedly be the cause of diarrhoea and it would be important to extend bacteriological identification in this line as well as in all cases of gastroenteritis of unknown aetiology.

## Introduction

The aquatic environment harbours *Vibrio cholerae* and this species has more than 200 serogroups categorized by O-polysaccharide specificity*. V. cholerae* O1 is the prominent serogroup causing epidemic or pandemic cholera, but infections due to non-O1 *V. cholerae* are on the rise and cannot be neglected [1]. Serogroups apart from O1 and O139 are designated as non-O1/non-O139 *V. cholerae*. They are found in coastal and marine environments causing cholera-like symptoms and other extraintestinal infections [2]. Generally, the non-O1/non-O139 vibrios carry virulence factors other than cholera toxin, such as heat-stable enterotoxin (Stn), haemolysin (HlyA), repeat in toxin (RTX) and the type III secretion system (T3SS), which may play major roles in causing infections [1]. Here, we report a case of non-O1/non-O139 *V. cholerae* wherein the aetiology was proved to be *V. cholerae* O6. According to a literature survey and to the best of our knowledge, the *V. cholerae* O6 isolated in the present study is the first to be reported from Karnataka state in South India.

## Case report

A 27-year-old female with acute gastroenteritis was admitted to Kasturba Hospital, Manipal, India. She was suffering from lupus nephritis class IV and on a NIH (National Institute of Health) protocol for 6 months. The patient was on a treatment regime with four cycles of cyclophosphamide (700 mg for 2 weeks) and she was due to receive the fifth pulse of cyclophosphamide. On admission, she had complaints of loose stools, vomiting, abdominal pain and fever for 1 day. Stools passed were watery with no mucus and blood. She had a history of oral candidiasis, upper respiratory tract infection and leucopenia.

## Investigations

Renal biopsy and histopathological immunofluorescent evaluation showed diffuse global proliferative lupus nephritis – class IV. Antinuclear antibody (ANA) detection by both immunofluorescence and immunoblot techniques showed a strong positive reaction. Based on these tests, the patient was diagnosed as lupus nephritis class IV. On examination, the patient’s vital signs were normal with a pulse rate of 84 beats min^−1^, a respiratory rate of 16 breaths min^−1^ and blood pressure of 130/70 mmHg. No abnormalities were detected in the cardiovascular, central nervous and respiratory systems. The patient's abdomen was soft, non-tender and there was no organomegaly. The patient was then put on intravenous hydration.

## Diagnosis

The stool microscopy showed no eggs or cysts. A faecal sample was then enriched in selenite F broth for 18 h and alkaline peptone water for 6 h, followed by inoculation on blood agar, MacConkey's agar, hektoen enteric agar and thiosulfate-citrate-bile salts-sucrose agar, and incubation at 37 °C for 18–24 h. The growth was processed for isolation of bacterial enteric pathogens viz. *Shigella* spp., *Salmonella* spp., *Vibrio* spp. and diarrhoeagenic *E. coli*, following World Health Organization guidelines. Stool culture was found to be positive for *V. cholerae* by standard biochemical tests. The bacterial isolate was confirmed as *V. cholerae* by using the Vitek 2 compact system (bioMérieux). Antimicrobial drug susceptibility assay was performed using the disc diffusion method (Becton Dickinson), according to Clinical and Laboratory Standards Institute guidelines. The isolate was sensitive for the antibiotics tested (Table 1). The *V. cholerae* did not show any agglutination with serogroups O1 or O139. The isolate was further sent to the National Institute of Cholera and Enteric Diseases (NICED), Kolkata, India, where it was confirmed as *V. cholerae* by *omp*W PCR [3] and serotyped as O6. The *omp*W PCR was used for the identification of the *V. cholerae* and to detect virulence factors. The *V. cholerae* O6 identified in this study was further subjected to simplex and multiplex PCR assays to detect the presence of virulence genes by using published methods [4]. In the multiplex PCR-based assay, a 301 bp amplicon of *ctxA* (which encodes subunit A of cholera toxin) and a biotype-specific *tcpA* allele (which encodes TcpA) were detected simultaneously. Simplex PCR assays were performed for the detection of *ompW*, *hlyA* and *rtxA*. The *vcsC2*, *vcsN2*, *vspD* and *vcsV2* genes were used as target loci for PCR-based amplification for the detection of the TTSS cluster in this *V. cholerae* non-O1, non-O139 strain. The *V. cholerae* O6 isolate lacked *ctxA* (classical/El Tor), *rtxA*, *stn* and *tcpA* (classical/El Tor) genes. It was found to harbour some virulence genes, such as haemolysin (*hlyA*) and structural genes of the T3SS (Table 2), which could play a vital role in the cytotoxicity and invasion required for the infection process.

**Table 1.** Antimicrobial-susceptibility testing of the *V. cholerae* O6 isolate

**Antibiotic**	**Sensitivity**
Ampicillin (AMP)	S
Azithromycin (AZM)	S
Cefotaxime (CTX)	S
Ceftazidime (CAZ)	S
Ceftriaxone (CRO)	S
Ciprofloxacin (CIP)	S
Doxycycline (D)	S
Erythromycin (E)	S
Gentamicin (G)	S
Meropenem (MEM)	S
Nalidixic acid (NA)	S
Neomycin (N)	S
Norfloxacin (NOR)	S
Ofloxacin (OFX)	S
Streptomycin (S)	S
Tetracycline (TE)	S
Trimethoprim/sulfamethoxazole (SXT)	S

S, Sensitive.

**Table 2.** Molecular characterization of the *V. cholerae* O6 isolate

**Virulence gene**							
***ctxA***	***tcpA* (classical)**	***tcpA* (El Tor)**	***rtxA***	***hlyA***	***ompW***	**NAG ST**	**T3SS structural genes**
−	−	−	−	+	+	−	+

## Treatment

Oral ciprofloxacin (250 mg) was prescribed twice daily for 5 days. Hand hygiene and water hygiene were explained to the patient, and she was discharged.

## Outcome and follow-up

After treatment, the patient was symptomatically better, no intestinal pathogens were reported from her repeat stool microbiological investigations.

## Discussion

Non-O1/non-O139 *V. cholerae* serogroups are reported to have caused sporadic diarrhoea as well as outbreaks of gastroenteritis [5, 6]. The mode of transmission for cholera infections is strongly related to water exposure or ingestion of undercooked and raw food [7]. The coastal belt in rural Karnataka, Manipal, located in southern India, is an exceptional habitat where the growth of *V. cholerae* flourishes because of the high salinity of the water sources. The *V. cholerae* O6 isolated was found to be sensitive to all antibiotics tested viz. ampicillin, ciprofloxacin, cotrimoxazole and tetracycline, which was found to be in concordance with other studies done. PCR in this study detected significant virulence genes, such as *hlyA*, *ompW* and T3SS genes, which are said to encode the virulence of *V. cholerae* non-O1, non O139 strains. A study carried out at the National Institute of Cholera and Enteric Diseases (NICED), India, during the years 2002–2010 reported some of the serogroups of non-O1/non-O139 *V. cholerae* associated with diarrhoea (O6, O11, O37, O34, O35, O59 and O97). A study carried out by Theophilo *et al*., in Brazil, reported the isolation of three *V. cholerae* O6 from suspected cholera patients and all were positive for the *zot* gene, which encodes an enterotoxin that increases intestinal permeability, leading to the disassembly of the intercellular tight junctions [8]. A study from China during the years 2001 to 2009 detected the serogroups O9, O38 and O76 [9].

Gastroenteritis caused by non-O1/non-O139 *V. cholerae* can be mild to severe, with stools watery in consistency but less frequently associated with blood [1, 10]. The serogroups O10, O11, O12 and O144 seem to be more often associated with disease, despite the absence of virulence factors, indicating that these serogroups have a mode of pathogenesis different from that of toxigenic *V. cholerae* [1, 4]. Heterogeneous factors are responsible for the virulence of this bacteria and type III secretion facilitates colonization. The enteroinvasive nature of some non-O1/non-O139 vibrios is because of two major components, haemagglutinin protease and haemolysin, present in the *V. cholerae* [2]. While a few of these strains produce cholera or cholera-like toxin, the majority of them lack these virulence genes. However, they secrete several other extracellular products, such as non-O1/non-O139 vibrio-specific heat-stable toxin, haemagglutinin, Shiga-like toxin and a thermostable direct haemolysin, which play a significant role in causing the infection [5].

Immunosuppressive drugs like cyclophosphamide given for lupus nephritis can depress white blood cell counts and increase the risk of infection. Vital information in this case presentation is that although cyclophosphamide is the most commonly used drug in autoimmune disease and cancer, it can cause dysbiosis of gut microbiota linked to various human diseases and can suppress the immune function. The prolonged cyclophosphamide treatment given in the present case might have suppressed the immunity of the patient, thereby enabling the non-O1/non-O139 *V. cholerae* to cause acute gastroenteritis. In conclusion, this case provides relatively comprehensive information to clinicians and microbiologists that when patients are on immunosuppressive drugs, there is a continual threat of contagious and opportunistic pathogens causing systemic infections. The constant changes in the characteristics of *V. cholerae*, either in the serogroups predominating in an outbreak or its virulence, need close monitoring for the treatment and control of the disease. Although many clinical laboratories do not give importance to non-O1/non-O139 vibrios, these serogroups should be taken into consideration when a suspected cholera case is encountered. Studying their pattern also provides useful information regarding the evolution of this pathogen. The diversity of serogroups causing cholera may be a survival advantage to the pathogen in the wake of a less-susceptible host. These strains, along with multidrug-resistant clones, may form a group of emerging pathogens in the future. However, the mechanisms of new epidemic strains and the ecology supporting such a development require much further understanding.
